# Time-resolved resonant soft X-ray scattering combined with MHz synchrotron X-ray and laser pulses at the Photon Factory

**DOI:** 10.1107/S1600577522008724

**Published:** 2022-10-06

**Authors:** Ryo Fukaya, Jun-ichi Adachi, Hironori Nakao, Yuichi Yamasaki, Chihiro Tabata, Shunsuke Nozawa, Kouhei Ichiyanagi, Yuta Ishii, Hiroyuki Kimura, Shin-ichi Adachi

**Affiliations:** a Institute of Materials Structure Science, High Energy Accelerator Research Organization, Tsukuba, Ibaraki 305-0801, Japan; b Graduate University for Advanced Studies (SOKENDAI), Tsukuba, Ibaraki 305-0801, Japan; c Research and Services Division of Materials Data and Integrated System, National Institute for Materials Science, Tsukuba, Ibaraki 305-0044, Japan; d Institute for Integrated Radiation and Nuclear Science, Kyoto University, Kumatori, Osaka 590-0494, Japan; e Department of Physics, Tohoku University, Sendai, Miyagi 980-8578, Japan; f Institute of Multidisciplinary Research for Advanced Materials, Tohoku University, Sendai, Miyagi 980-8577, Japan; University of Tokyo, Japan

**Keywords:** time-resolved X-ray experiments, resonant soft X-ray scattering, high-repetition-rate data acquisition, photoinduced phase transitions, ultrafast material science

## Abstract

A picosecond pump–probe resonant soft X-ray scattering measurement system has been developed at the Photon Factory storage ring for highly efficient data collection. A high-repetition-rate high-power compact laser system has been installed to improve efficiency via flexible data acquisition to a sub-MHz frequency in time-resolved experiments. Data are acquired by gating the signal of a channel electron multiplier with a pulse-counting mode capable of discriminating single-bunch soft X-ray pulses in the dark gap of the hybrid operation mode in the storage ring. The photoinduced dynamics of magnetic order for multiferroic manganite SmMn_2_O_5_ are clearly demonstrated by the detection of transient changes in the resonant soft X-ray scattering intensity around the Mn *L*_III_- and O *K*-edges.

## Abstract 2

A picosecond pump–probe resonant soft X-ray scattering measurement system has been developed at the Photon Factory storage ring for highly efficient data collection. A high-repetition-rate high-power compact laser system has been installed to improve efficiency via flexible data acquisition to a sub-MHz frequency in time-resolved experiments.

## 1. Introduction

Controlling physical properties such as conductivity, electricity and magnetism on an ultrafast timescale using femtosecond (fs) and picosecond (ps) laser pulses has been an important strategy in developing the next generation of ultrafast switching and communication devices and has led to new developments and understanding in material science. From this viewpoint, new functionalities have emerged from photonically perturbing the charge, spin, orbital and lattice degrees of freedom in strongly correlated electron systems (Basov *et al.*, 2017 ▸; Koshihara *et al.*, 2022 ▸). Photoinduced quantum phase transitions in a strongly correlated electron system are typically triggered by a local change in the electronic states, leading to a cooperative interaction between electron and lattice degrees of freedom on the fs and ps timescales. These microscopic changes develop into macroscopic quantum phase transitions with structural, orbital and elastic cooperative effects on a nanosecond (ns) timescale, resulting in a variety of physical properties (Collet *et al.*, 2003 ▸; Ichikawa *et al.*, 2011 ▸; Bertoni *et al.*, 2016 ▸). Therefore, observing the process of microscopic-to-macroscopic change is essential to understanding the mechanism of photoinduced quantum phase transitions. Time-resolved measurements using a pump and probe are useful for capturing transient phenomena on ultrafast timescales. X-ray diffraction/scattering is a probing method that reveals not only the lattice structure but also the electronic order characteristics of physical properties by tuning the photon energy of the X-ray pulse to the absorption edge of the constituent elements. In particular, resonant soft X-ray scattering (RSXS) can probe the 3*d* electronic state directly using the *L*_II,III_-edge (2*p* → 3*d* transition) of transition metal ions; thus, the signal of the resonant magnetic scattering can be well observed. Moreover, the RSXS signal around the oxygen *K*-edge reflects the localized and itinerant character of an oxygen ion through 2*p*–3*d* orbital hybridization between transition metal and oxygen ions (Nakao *et al.*, 2018 ▸). Hence, time-resolved RSXS has a great advantage for studying ultrafast dynamics of electronic order in strongly correlated electron systems.

Synchrotron radiation emitted from a storage ring with a MHz repetition rate is available for pump–probe experiments using picosecond pulses in the soft X-ray photon energy regions. In time-resolved measurements, however, the average X-ray photon flux is reduced generally because of the thinning out of the synchrotron X-ray pulse train in accordance with the repetition rate of the pumping laser. Because a Ti:sapphire regenerative amplifier operating at a fixed repetition rate of a few kHz is typically used as a pumping laser, X-ray pulses with fixed repetition rates two or three orders lower than those in static experiments are used in time-resolved experiments. Furthermore, it is difficult to easily tune the data acquisition frequency to suit signal intensities and sample characteristics. Therefore, the number of synchrotron facilities where photon-hungry time-resolved RSXS experiments can be conducted has been limited (Ehrke *et al.*, 2011 ▸; Doering *et al.*, 2011 ▸; Holldack *et al.*, 2014 ▸).

With recent developments in laser technology, MHz-repetition-rate lasers with high pulse energies comparable with that of a Ti:sapphire regenerative amplifier have become commercially available. Combining MHz synchrotron X-rays and high-repetition-rate laser pulses improves the average probing RSXS signal in time-resolved measurements. Furthermore, making the laser system easily portable makes it possible to construct a time-resolved X-ray measurement system at various beamlines for probing a range of X-ray photon energies. In this study, we installed a high-repetition-rate high-power pumping laser system for time-resolved RSXS experiments on the BL-16A and BL-19B beamlines at the Photon Factory of the High Energy Accelerator Research Organization, Japan. Here, we briefly report the setup of this time-resolved RSXS measurement system adopting a hybrid filling operation of the storage ring, and we demonstrate the transient RSXS responses related to the photoinduced dynamics of the magnetic order and 2*p*–3*d* orbital hybridization between transition metal and oxygen ions in multiferroic materials among strongly correlated electron systems.

## 2. Experimental setup

### 2.1. Hybrid filling mode at the Photon Factory storage ring

**Figure 1:**
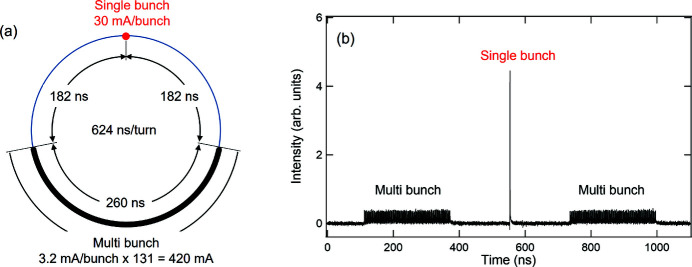
(*a*) Schematic of the filling pattern of the hybrid mode at the Photon Factory ring. (*b*) Time-domain intensity of soft X-ray pulses at 775 eV in the hybrid filling mode measured using the MSM-PD on BL-19B.

The Photon Factory storage ring is operated at an energy of 2.5 GeV and total current of 450 mA with a radio-frequency (RF) master clock of 500.1 MHz (= *f*_RF_) and two types of bunch-filling patterns, namely multi-bunch and hybrid bunch patterns. The multi-bunch mode has typically 250 bunches with a bunch-by-bunch interval of 2 ns. Meanwhile, the hybrid mode has a multi-bunch train with 131 bunches, a total current of 420 mA (3.2 mA per bunch), and a high-current single bunch at 30 mA, as shown in Fig. 1 ▸(*a*). The gap between the single bunch and multiple bunches is set at 182 ns, and the interval of the single-bunch revolution frequency (*f*_rev_ = *f*_RF_/312 = 1.6 MHz) is 624 ns. Fig. 1 ▸(*b*) shows the time structure of the soft X-ray pulse in the hybrid mode measured using an ultrafast metal–semiconductor–metal photodetector (MSM-PD), reflecting the hybrid filling pattern of the single and multi-bunches. A high-intensity single soft X-ray pulse is observed in the dark gap of the multiple bunches. The root-mean-square (r.m.s.) bunch length of the single-bunch component is approximately 45 ps as measured using a streak camera (Takai *et al.*, 2010 ▸; Yamamoto *et al.*, 2020 ▸). In time-resolved experiments, it is necessary to pump the sample in accordance with the single-bunch timing and then selectively detect only the X-ray signals from the corresponding single bunches.

### 2.2. Pump–probe resonant soft X-ray scattering measurement system

**Figure 2:**
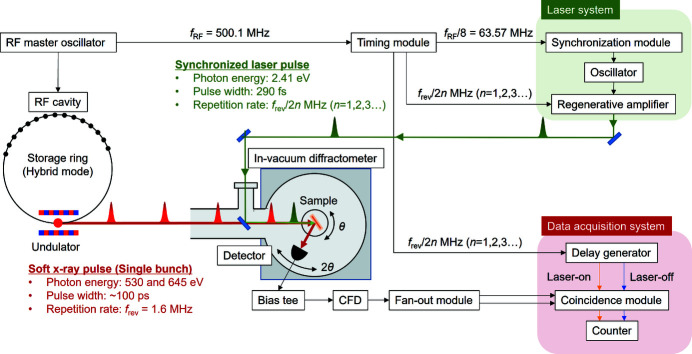
Schematic overview of the pump–probe RSXS measurement system. The RF master oscillating signal, which is the reference signal for the storage ring, is separated into three timings by the timing module to synchronize the oscillator, the laser emission of the regenerative amplifier and the data acquisition system. The laser beam introduced into the vacuum path irradiates the sample almost coaxially with the soft X-ray beams. In this study, the photon energy of the laser is 2.41 eV (515 nm) and that of the X-rays is approximately 530 and 645 eV corresponding to the O *K*- and Mn *L*_III_-edges, respectively. A constant-fraction discriminator is abbreviated as CFD.

Fig. 2 ▸ shows an overview of the experimental setup for the pump–probe RSXS measurements at the Photon Factory. An in-vacuum two-circle (θ–2θ) diffractometer, where the horizontal plane is the scattering plane, is used in the pump–probe RSXS experiment (Nakao *et al.*, 2014 ▸). In this diffractometer, a He-flow cryostat that realizes a cold head temperature of <10 K is installed. The sample is cooled by thermal contact from the sample holder attached to the cold head of the cryostat. The detector in the pump–probe RSXS experiment is a channel electron multiplier (CEM) (Photonis, model 5901 with a KBr coating), which has a high gain and fast response to the pulse signals with a pulse-counting mode. It is therefore possible to detect the RSXS signal from the single bunch via electronic gating without mechanically selecting the X-ray pulse using an X-ray chopper. Furthermore, the detector is insensitive to the pumping laser light, which has lower photon energy than X-rays; thus, the huge background from the laser light can be excluded without using optical filters in the detector. The single-bunch signal obtained from the CEM through a bias tee (Tektronix, PSPL5530B) is discriminated from the background noise by a constant-fraction discriminator (ORTEC, model 934). The signal is then divided into two signals by a fan-out module (Technoland, N-TY202) and the two signals are recorded in integration mode with a coincidence module (LeCroy Research Systems, model 622) through an electronic gate process triggered by a digital delay pulse generator (Stanford Research Systems, DG645). The data acquisition scheme is described in the next subsection.

A MHz-order high-repetition-rate and high-power laser system is required to use effectively the single-bunch soft X-ray pulse emitted routinely at 1.6 MHz in the hybrid mode and produce the photoexcited state of the sample with sufficient laser intensity. In the pump–probe RSXS measurement, we used an ytterbium-doped potassium–gadolinium tungstate (Yb:KGW) medium diode-pumped femtosecond laser system (Light Conversion, PHAROS). The repetition rate and pulse duration can be tuned from a single shot to 1 MHz and from 290 fs to 10 ps, respectively. The maximum pulse energy is 200 µJ and the maximum average power is 20 W. The wavelength of the fundamental light is 1030 nm. Second (515 nm) and third (343 nm) harmonics are available when an automated harmonic generator is used. The laser and synchrotron radiation are synchronized by phase locking the oscillator at 63.513 MHz (= *f*_RF_/8) to seed the regenerative amplifier, using a frequency synchronization module (Menlo Systems, RRE-SYNCRO). The timing jitter of the synchronization module is <30 fs when using a harmonic RF signal and <500 fs when using a fundamental RF signal. A timing module controls the repetition rate of emission of the amplified laser pulse with revolution of the single-bunch repetition frequency [*f*_rev_/2*n* (*n* = 1, 2, 3…)] and the laser timing delay on the basis of the RF master frequency. The timing module has a phase shifter, phase counter and frequency divider (Candox Systems, Trigger Clock delay module). There is concern regarding the laser-induced heating and damage to the sample from irradiation with high-repetition-rate laser pulses. Therefore, the repetition rate is adjusted in accordance with the experimental conditions. The overall timing jitter between the laser and synchrotron radiation mainly depends on the jitter of the timing module (<5 ps) because this jitter is ten times that of the synchronization module. The synchronized laser beam is introduced into the vacuum path passing through the soft X-ray beam and it then irradiates the sample in the in-vacuum diffractometer almost coaxially with the soft X-ray beam. The laser head is installed near the in-vacuum diffractometer on a relatively small optical table (1200 mm × 600 mm). The laser system is portable, and the pump–probe measurement system can thus be constructed even in beamlines with limited space.

### 2.3. Data acquisition scheme

**Figure 3:**
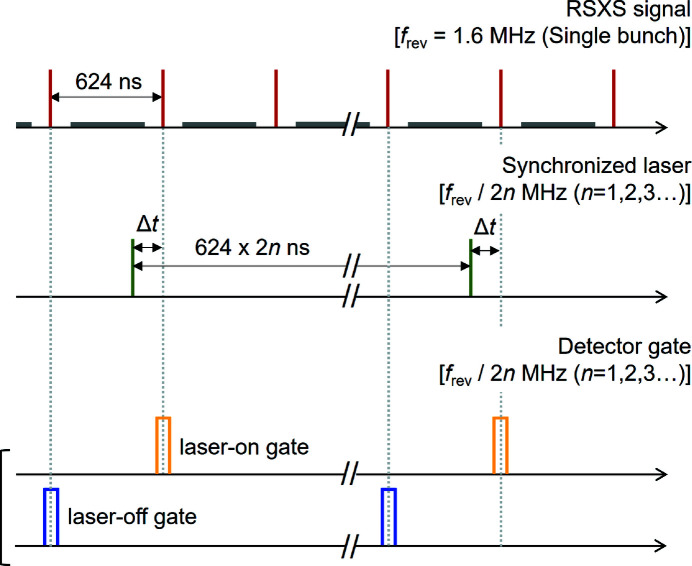
Timing chart of the data acquisition process. The upper and middle pulse trains show the timing of the detected RSXS signal with the hybrid bunch pattern and of the synchronized laser pulse. The single and multiple bunches including the RSXS signal are indicated by the red and grey pulses, respectively. The laser delay time is denoted Δ*t*. The lower two gating signals indicate the laser-on gate timing (upper) and laser-off gate timing (lower) of the pumping laser pulses.

The single-bunch RSXS signals corresponding to the synchronized laser timing are extracted from the signal collected by the CEM with the hybrid bunch pattern via electronic gate processing. A timing chart of the RSXS signals, synchronized laser pulses and detector gates is shown in Fig. 3 ▸. The repetition rate of the synchronized laser is set at double the number of dividing frequencies of the single-bunch frequency; *i.e.* *f*_rev_/2*n* (*n* = 1, 2, 3…), because the low-frequency noise components are removed by detecting the difference between the laser-on and laser-off signals for each laser pulse and avoiding laser heating. Using the coincidence technique, two single-bunch RSXS signals with different timings are collected simultaneously at the same frequency as the laser pulse; one is the pulse after the laser delay time Δ*t* and the other is the previous single-bunch timing. The signal at the laser-off gate timing is supposed to be the ground-state signal before the laser pumping because enough time has elapsed since the laser excitation timing. The trigger timings corresponding to the laser-on and laser-off single-bunch X-ray pulses are generated and tuned by the digital delay generator. To subtract the signal from the multi-bunches, the width of both electronic gates is set at approximately 20 ns within the dark gap between the single and multiple bunches. Although the repetition rate of the synchronized laser strongly depends on the sample conditions, we can collect the data efficiently up to a repetition rate of half of *f*_rev_ (800 kHz) in the constructed time-resolved RSXS measurement system.

### 2.4. Sample and experimental conditions

*R*Mn_2_O_5_ (*R* = rare-earth) compounds are multiferroic materials coupled with antiferromagnetism and ferroelectricity that have attracted attention because a variety of magnetoelectric effects have been exhibited by changing the *R* ions (Hur *et al.*, 2004 ▸; Fukunaga *et al.*, 2009 ▸). Among the *R*Mn_2_O_5_ family, SmMn_2_O_5_ has a high electric polarization and a unique orientation of the magnetic moments (Ishii *et al.*, 2016 ▸, 2018 ▸; Yahia *et al.*, 2017 ▸). An RSXS experiment around the Mn *L*_II,III_- and O *K*-edges of *R*Mn_2_O_5_ provides important information not only on the ordering of the Mn magnetic moment coupled with the electronic state but also on the spin polarization of oxygen ions via 2*p*–3*d* orbital hybridization of oxygen and Mn ions (Partzsch *et al.*, 2011 ▸; de Souza *et al.*, 2011 ▸; Huang *et al.*, 2016 ▸; Ishii *et al.*, 2018 ▸). We demonstrate the transient dynamics of the magnetic ordering and electronic polarization in SmMn_2_O_5_ by high-repetition-rate pump–probe RSXS measurement around the Mn *L*_III_- (645 eV) and O *K*-edges (530 eV) on BL-16A at the Photon Factory of the High Energy Accelerator Research Organization. This beamline provides an X-ray photon flux of approximately 10^11^ photons s^−1^ with a spot size of 500 µm (horizontal) and 100 µm (vertical). A single crystal of SmMn_2_O_5_ was grown using the PbO–PbF_2_ flux method (Wanklyn, 1972 ▸). The lattice parameters were *a* = 7.425 Å, *b* = 8.596 Å and *c* = 5.672 Å at room temperature (Ishii *et al.*, 2016 ▸). For the experiments, a sample with a surface normal of (1 0 0) was prepared by polishing. The dielectric and magnetic phase diagram for SmMn_2_O_5_ has been obtained by measurements of dielectric properties, magnetic susceptibilities, resonant hard X-ray scattering and powder neutron scattering (Ishii *et al.*, 2016 ▸; Yahia *et al.*, 2017 ▸). Several anomalies of the magnetic susceptibility have been observed around 26, 28, 34 and 44 K, which are ascribed to changes in both Sm and Mn magnetic moments (Ishii *et al.*, 2016 ▸, 2018 ▸). The excitation wavelength was used at the second-harmonic light (515 nm), whose photon energy mainly corresponds to the charge transfer excitation between Mn and O ions in *R*Mn_2_O_5_ (Moskvin & Pisarev, 2008 ▸). The spot size was set at approximately 1 mm (horizontal) and 1 mm (vertical), and an excitation fluence of less than 100 µJ cm^−2^ was incident on the sample. The repetition rate of the synchronized laser and detector gates was set at 22.9 kHz to avoid an increase in temperature of the sample through laser heating and to collect the data as efficiently as possible.

## 3. Experimental results

**Figure 4:**
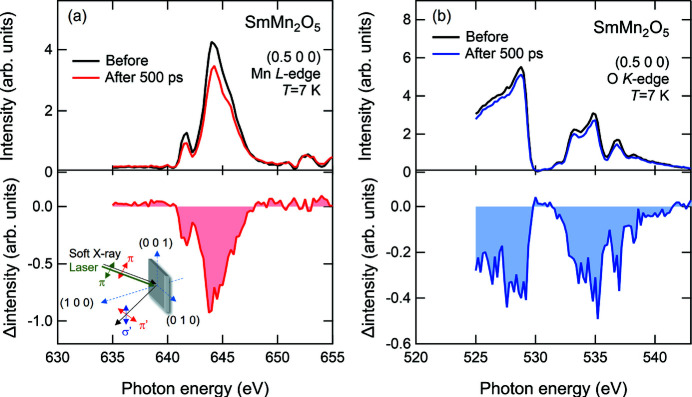
Energy spectra of the (0.5 0 0) diffraction peaks at 7 K before and after laser pumping at 500 ps around the (*a*) Mn *L*_III_- and (*b*) O *K*-edges measured with a 22.9 kHz repetition rate. The excitation fluence is 60 µJ cm^−2^. Both lower spectra in (*a*) and (*b*) are the difference spectra of measurement before and after laser pumping. The inset of (*a*) is a schematic of the experimental geometry of the soft X-rays and laser.

Figs. 4 ▸(*a*) and 4(*b*) show, respectively, the transient energy spectra of the (0.5 0 0) diffraction peaks at 7 K before and after laser pumping around the Mn *L*_III_- and O *K*-edges for SmMn_2_O_5_. A schematic of the experimental geometry of the incident and scattered soft X-rays is shown in the inset of Fig. 4 ▸(*a*). The polarization of both the soft X-rays and laser is in the horizontal (π) configuration. The intensity at the (0.5 0 0) reflection was detected without analyzing the polarization and hence included both horizontally (π′) and vertically (σ′) polarized components. Spectra were collected before and after laser pumping at the laser-off and laser-on gate signals with Δ*t* = 500 ps, as described in Section 2.3. Both spectra before the laser pumping around the Mn *L*_III_- and O *K*-edges acquired in the electronic gate processing well reproduce the static spectra in previous reports obtained via the usual detection method, reflecting the magnetic ordering in the commensurate magnetic phase below 26 K (Ishii *et al.*, 2018 ▸). After the laser pumping, the spectral intensities around both edges decrease according to the spectral shapes, as shown by the difference spectra in Figs. 4 ▸(*a*) and 4(*b*). These spectral changes are direct signatures of the melting of the antiferromagnetic order with the (0.5 0 0) propagation vector.

**Figure 5:**
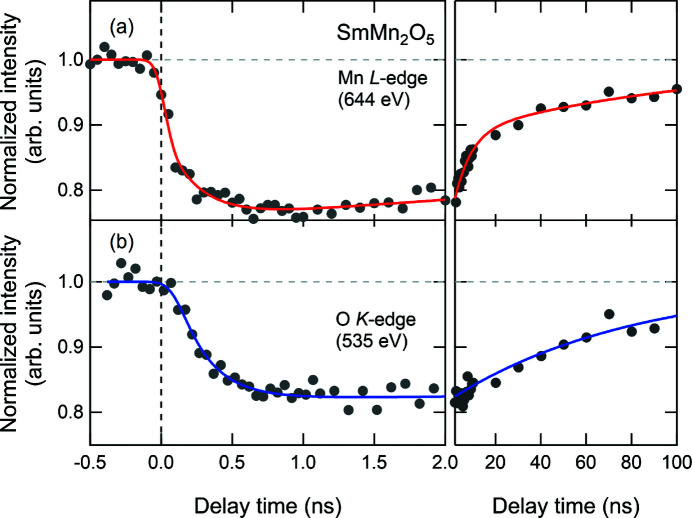
Time profiles of the (0.5 0 0) diffraction peak intensities (*a*) at 644 eV at the Mn *L*_III_-edge and (*b*) at 535 eV at the O *K*-edge. The time intervals are changed after 2 ns.

Figs. 5 ▸(*a*) and 5(*b*) presents plots of the time-dependent changes in the normalized RSXS intensities before and after laser pumping around the Mn *L*_III_-edge at 644 eV and around the O *K*-edge at 535 eV. The intensity changes were probed at the maximum of the difference spectra of measurements before and after laser pumping for the respective diffraction peaks. The intensities are clearly reduced after the laser pumping at time zero, strongly suggesting that the transient response is induced by the photoexcitation and by the dynamics around the Mn *L*_III_-edge, which are similar to those of La_0.5_Sr_1.5_MnO_4_, exhibiting antiferromagnetic order (Ehrke *et al.*, 2011 ▸). However, it seems that the initial decrease around the O *K*-edge is slightly slower than that around the Mn *L*_III_-edge, which responds to the photoexcitation within the time resolution. Moreover, a difference in decay dynamics of several tens of nanoseconds is observed. In quantitative analysis, we assume that the observed time profiles comprise two components, a fast decay component and a slow decay component, and that their total intensity *I*(*t*) is

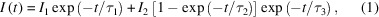

where the first term denotes the fast component, which responds to the time resolution and then decays with a time constant τ_1_, and the second is the slow component with changing time τ_2_ and slow decay time τ_3_. *I*_1_ and *I*_2_ are the component intensities. The spectra were fitted with a convolution between *I*(*t*) and the Gaussian instrument function with a time resolution of approximately 57 ps r.m.s. The fitting analysis shows that the time profiles in Figs. 5 ▸(*a*) and 5(*b*) can be well reproduced. The fitting parameters τ_1_, τ_2_ and τ_3_ are listed in Table 1 ▸. The time profile around the Mn *L*_III_-edge contains two different decay components with decay times of approximately 7 and 100 ns, corresponding to the fast and slow components, respectively. However, the profile around the O *K*-edge can be well reproduced with the fitting function only for the slow component, which responds approximately 200 ps after the laser pumping. These differences may reflect that the ferroelectricity in SmMn_2_O_5_ is due to the magnetically driven lattice distortion rather than the electronic polarization caused by the charge transfer through orbital hybridization between O and Mn ions (Ishii *et al.*, 2020 ▸). Although the exact origin of the two components is presently unclear, further experimental and theoretical studies may reveal the transient contribution to the ferroelectricity of the photoinduced melting of the antiferromagnetic ordering. The experimental results show the effectiveness of using the constructed pump–probe RSXS setup in a feasible acquisition time at a 22.9 kHz repetition rate, even though the RSXS signal around the O *K*-edge is weak generally. The improvement in the flexible data acquisition to the high repetition frequency for transient RSXS signals makes it possible to directly detect the transient electronic order and transient electronic coupling between metal and oxygen ions in strongly correlated transition metal oxide systems.

## 4. Summary

We implemented a high-repetition-rate pump–probe RSXS measurement system in the Photon Factory storage ring. The use of single-bunch soft X-ray pulses in hybrid operation mode and a repetition-rate-tunable high-power compact laser system improved the experimental efficiency through data acquisition with optimal tuning of the repetition rate (22.9 kHz) for time-resolved RSXS measurements on various beamlines with an in-vacuum diffractometer. We conducted a pump–probe RSXS experiment for multiferroic manganite around the Mn *L*_III_- and O *K*-edges and collected the transient RSXS signal over several hours. Further study is currently in progress. Performing high-repetition-rate pump–probe soft X-ray experiments using synchrotron radiation is a new strategy for studying transient phenomena on a picosecond-to-nanosecond timescale induced by photoexcitation.

## Figures and Tables

**Table 1:** Fitting parameters τ_1_, τ_2_ and τ_3_ obtained using the time-dependent function *I*(*t*)

Absorption edge	τ_1_ (ns)	τ_2_ (ns)	τ_3_ (ns)
Mn *L*_III_	7.32 ± 1.08	0.24 ± 0.04	113.5 ± 19.4
O *K*	–	0.22 ± 0.03	80.8 ± 6.22
